# Escape from quicksand: illicit drug use among youth in southeast Asia

**DOI:** 10.1016/j.lansea.2023.100175

**Published:** 2023-03-08

**Authors:** The Lancet Regional Health – Southeast Asia

Illicit drug use is an ongoing issue in southeast Asia. The Golden Triangle, an area where porous borders of Laos, Myanmar, and Thailand meet, is a major source for the stimulant and addictive drug, methamphetamine in southeast Asia. Massive illicit production from this region has increased the purity and notoriously lowered the price of methamphetamine, which in turn has made methamphetamine more affordable to youth. In 2021, more than 1 billion methamphetamine tablets were seized from east and southeast Asia. Youth—on a path to develop emotional maturity in adulthood—show more risky behaviours and are prone to illicit drug use. Compared with previous generations, the proportion of young people using illicit drugs is increasing. Among all people who use cannabis globally, the majority (42%) are aged 17–24 years. *Yaba*, a mixture of methamphetamine and caffeine, is mainly produced in Myanmar and is very popular in many countries in southeast Asia, such as Thailand and Bangladesh. Thai youth use *Yaba* to improve academic performance, make leisure activities more fun, and escape the loneliness and isolation associated with city life.

In Thailand, one could legally possess 15 methamphetamine pills before February 2, 2023. However, on October 6, 2022, a policeman in Thailand—suspended from duty for possessing methamphetamine—conducted a mass shooting at a daycare centre leading to 37 deaths, including 24 children. This incident was a driving force for the Health Ministry of Thailand to bring out stricter regulation by which an individual possessing more than one pill of methamphetamine would be labelled as a drug distributor. Unfortunately, the loss of several lives was required for the Thai Government to finally see the problem and tighten the grip on methamphetamine.

Thailand was the first Asian country to legalise cannabis in June, 2022, and medical cannabis in December, 2018. Cannabis consumption has negative effects, such as transient psychosis, depression, and schizophrenia. However, similarly to alcohol, it brings revenue. The cannabis market in Thailand is expected to grow to 21 billion Baht (US$661 million) by 2024, owing to tourism potential. Except at Thai government-approved outlets, recreational use of cannabis is technically banned. Additionally, the authorities need to randomly check products sold for the legal limit of 0.2% tetrahydrocannabinol, a procedure which is expensive and hence, a barrier to effective regulation of cannabis. The comment by Yimsaard and colleagues discusses the importance of studying the impact of cannabis legalisation on youth in Thailand, specifically cannabis-related driving and accidents. The authors stress that the primary goal should be public health protection by promoting public awareness on the risk of cannabis use. Data from Canada, Uruguay, and USA show that cannabis legalisation over longer periods leads to an increase in potency (tetrahydrocannabinol concentration) and a reduction in price and risk perception, thus resulting in increased use by adolescents.

Illicit drugs and conflicts go hand in hand. Myanmar ranks second for opium production worldwide (after Taliban occupied Afghanistan), which is promoted by the military junta. Farmers residing in conflict zones are switching to opium farming. Economic disruption in Myanmar due to the military coup in February, 2021, and the COVID-19 pandemic has culminated in more people using illicit drugs. Shockingly, in some regions of Myanmar such as Kachin, many children above age 11 years are using injectable drugs.

Childhood adversities, such as physical, emotional, or sexual abuse and neglect have huge negative impact on an individual. Police cases related to drugs in the state of Kerala in India have skyrocketed with an increase of 333% during last year. Unsurprisingly, the main reasons cited were issues with parents and peer pressure. A cohort study in India by Fernandes and colleagues showed that males were more likely to consume tobacco, alcohol, or cannabis if they had experienced childhood adversities. Instead of dismissing students who used drugs, educational institutions should raise this issue with the police and authorities to counsel the parents and help youth to cope with stress. Drug abstinence accompanies the risk of a relapse overdose and needs to be monitored. A person with a history of drug misuse would become less tolerant and use of a similar amount of drug later might lead to an overdose. Sensitising the health professionals and police is also important to address these issues.

Media should aim to discourage depiction of drug use in a positive tone. Youth can be curious why celebrities are not prosecuted when casually discussing their experiences with drugs, which might lead to moral disengagement and illicit drug use among youth engaging in risky behaviour. Movie scenes involving substance misuse should have statutory warnings, which is not done for over-the-top media services in India.

Governments promoting drug use by justifying economic growth are trampling over the health of younger generations. Let raging hormones flow through the veins of youth, not illicit drugs. Parents and the society need to ensure that young adults have a future to look forward to, since they are the future.

